# Unraveling White Adipose Tissue Heterogeneity and Obesity by Adipose Stem/Stromal Cell Biology and 3D Culture Models

**DOI:** 10.3390/cells12121583

**Published:** 2023-06-08

**Authors:** Leandra S. Baptista, Karina R. Silva, Lara Jobeili, Lucile Guillot, Dominique Sigaudo-Roussel

**Affiliations:** 1 Numpex-bio, Campus UFRJ Duque de Caxias Prof Geraldo Cidade, Universidade Federal do Rio de Janeiro, Rio de Janeiro 25240005, Brazil; 2 Laboratory of Stem Cell Research, Histology and Embryology Department, Biology Institute, State University of Rio de Janeiro, Rio de Janeiro 20550900, Brazil; karina.pereira@uerj.br; 3 Teaching and Research Division, National Institute of Traumatology and Orthopedics, Rio de Janeiro 20940070, Brazil; 4 Laboratory of Tissue Biology and Therapeutic Engineering, University of Lyon, Claude Bernard University Lyon 1, CNRS, LBTI UMR 5305, 69367 Lyon, France; lara.jobeili@ibcp.fr (L.J.); lucile.guillot@ibcp.fr (L.G.); dominique.sigaudo-roussel@ibcp.fr (D.S.-R.); 5 Urgo Research Innovation and Development, 21300 Chenôve, France

**Keywords:** obesity, adipose tissue, adipose stem/stromal cells, 3D culture, spheroids, organoids

## Abstract

The immune and endocrine dysfunctions of white adipose tissue are a hallmark of metabolic disorders such as obesity and type 2 diabetes. In humans, white adipose tissue comprises distinct depots broadly distributed under the skin (hypodermis) and as internal depots (visceral). Depot-specific ASCs could account for visceral and subcutaneous adipose tissue properties, by regulating adipogenesis and immunomodulation. More importantly, visceral and subcutaneous depots account for distinct contributions to obesity and its metabolic comorbidities. Recently, distinct ASCs subpopulations were also described in subcutaneous adipose tissue. Interestingly, the superficial layer closer to the dermis shows hyperplastic and angiogenic capacities, whereas the deep layer is considered as having inflammatory properties similar to visceral. The aim of this focus review is to bring the light of recent discoveries into white adipose tissue heterogeneity together with the biology of distinct ASCs subpopulations and to explore adipose tissue 3D models revealing their advantages, disadvantages, and contributions to elucidate the role of ASCs in obesity development. Recent advances in adipose tissue organoids opened an avenue of possibilities to recreate the main cellular and molecular events of obesity leading to a deep understanding of this inflammatory disease besides contributing to drug discovery. Furthermore, 3D organ-on-a-chip will add reproducibility to these adipose tissue models contributing to their translation to the pharmaceutical industry.

## 1. Introduction

The immune and endocrine dysfunctions of white adipose tissue are a hallmark of metabolic disorders such as obesity and type 2 diabetes. These dysfunctions negatively impact on insulin sensitivity, metabolism, and promote local and systemic inflammation [1,2,3]. In humans, white adipose tissue comprises distinct depots broadly distributed under the skin (hypodermis) and as internal depots (visceral). The visceral depots are described as having inflammatory properties and are associated with metabolic disorders. The hypodermis or subcutaneous adipose tissue shows hyperplastic capacities besides being the preferential depot for mesenchymal stem/stromal cells known as adipose tissue-derived stem/stromal cells (ASCs). Depot-specific ASCs could account for visceral and subcutaneous adipose tissue properties by regulating adipogenesis and immunomodulation [4].

Subcutaneous adipose tissue can be divided into two main layers—superficial and deep—delimited by a conjunctive fascia. Recently, distinct ASCs subpopulations were also described in subcutaneous adipose tissue. Interestingly, the superficial layer closer to the dermis shows hyperplastic and angiogenic capacities, whereas the deep layer is considered as having inflammatory properties similar to visceral. These ASCs subpopulations have distinct contributions to subcutaneous adipose tissue physiology and may represent the key to the recovery of ASC properties in an inflammatory scenario [5].

Currently, the inability of traditional monolayer culture and animal models to reproduce molecular and cellular events of human tissues and organs is a consensus in the scientific literature. Three-dimensional cell culture models such as spheroids and organoids are increasingly used as models more faithful to the human cell physiology, creating a need for the development of functional adipose tissue 3D models [6].

The aim of this focus review is to bring the light of recent discoveries into white adipose tissue heterogeneity together with the biology of distinct ASCs subpopulations and to explore adipose tissue 3D models revealing their advantages, disadvantages, and contributions to elucidate the role of ASCs in obesity development. We further provide considerations on which methodological and technological advances may be necessary to improve obese adipose tissue in vitro modeling. This review may guide further studies in which both ASCs from subcutaneous and visceral depots and from ASCs subpopulations of distinct layers of subcutaneous adipose tissue may unravel cellular and molecular mechanisms as potential targets for the recovery of ASC and adipose tissue function in the context of obesity.

## 2. White Adipose Tissue

Knowledge of white adipose tissue physiology and metabolic dysfunctions has expanded significantly in recent decades. Once regarded as a simple inert energy storage tissue, white adipose tissue is now recognized as a biologically dynamic organ. More importantly, white adipose tissue has diffuse locations throughout the body as fat depots. Different physiologies, cell compositions, and functions are characterized for visceral and subcutaneous depots, and recently, for the different layers of the subcutaneous.

### 2.1. Subcutaneous and Visceral Depots and the Intrinsic Differences of Adipose Tissue-Derived Stem/Stromal Cells

White adipose tissue (WAT) acts primarily as a regulatory center for the homeostasis of the body’s energy metabolism. This is accomplished through the regulation of adipocyte lipid storage or release in response to the body’s energy demands, regulation of blood glucose levels due to the high insulin sensitivity of adipocytes [7], and through its secretory function [7,8]. The array of adipose secretory products, called adipokines, mediates inter-organ communication. This influences the metabolism and function of central and peripheral organs, including the immune system function [8,9].

WAT is one of the body’s pathways that, through remodeling of adipose tissue, allows adaptation to metabolic challenges posed by different external environmental changes, including food deficit, energy excess, stress, infection, or cold [10]. Crosstalk between different cell types composing the WAT stromal-vascular fraction (SVF) and adipocytes orchestrates the mechanisms of WAT remodeling [11]. Physiological remodeling that occurs during WAT expansion is characterized by angiogenesis, extracellular matrix remodeling, minimal inflammation, and adipocyte hyperplasia, through the recruitment of adipocyte precursors that provide capacity to store extra lipids during its adipogenic differentiation into small adipocytes (i.e., adipogenesis). The ability of WAT depots to expand through hyperplasia may be crucial for adequate lipid storage during WAT expansion and to avoid harmful ectopic lipid deposition in non-adipose tissues [12]. However, the chronic demand for excess energy storage that occurs during the development of obesity can trigger mechanisms of an unhealthy—or pathologic—WAT remodeling during its expansion, characterized by dysfunctional hypertrophic adipocytes, insufficient vascularization and hypoxia, fibrosis [10,11], infiltration of immune cells, and pro-inflammatory responses that contribute to tissue inflammation and subsequently insulin resistance [13,14,15].

**Figure 1:**
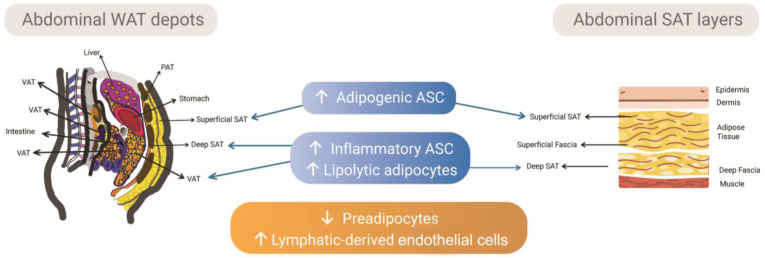
Abdominal WAT depots, differences in the SVF content, and their ASC and adipocytes’ functional profile. Major abdominal WAT in humans comprises subcutaneous and visceral depots (left image). VAT localizes around digestive organs. SAT has a superficial layer of small fat lobules that dwells below the skin, and a deep layer formed by large fat lobules (right image). These layers are separated by a conjunctive fascia (superficial fascia), from which emerges fibrous septae that encompass the fat lobules of the superficial SAT (right image). The functional profiles of ASC and in vitro-derived adipocytes are provided in blue boxes: ASCs from the superficial SAT are more adipogenic, while those from deep SAT and VAT are more inflammatory. The adipocytes derived from VAT and deep SAT have more lipolytic activity than those from the superficial SAT. The orange box shows the differences in the SVF content already described for VAT versus SAT. The SVF components vary according to the WAT depot: VAT has less preadipocytes and higher lymphatic-derived endothelial cells than SAT. SAT: Subcutaneous adipose tissue; VAT: Visceral adipose tissue; PAT: Preperitoneal adipose tissue; ASC: adipose-derived stromal/stem cells.

WAT depots with distinct expansion and remodeling patterns are widely distributed in the human body, comprising mainly subcutaneous and visceral depots. They have intrinsic biological differences [16] and differentially impact on obesity-induced metabolic complications: in obesity, visceral adipose tissue (VAT) is more associated with the risk to develop insulin resistance and type 2 diabetes than subcutaneous adipose tissue (SAT) [17]. These functional differences among WAT depots and their distinct contributions to obesity and its metabolic comorbidities are being attributed not only to differences in their SVF composition (i.e., frequency of mesenchymal stem cells, pre-adipocytes, endothelial progenitor cells, mature endothelium, and immune cells subtypes), but also to the functional diversity of adipocyte stem cells and progenitors cells that derive specialized adipocyte subtypes [4,18,19,20] (Figure 1).

We have previously shown that the SVF of obese abdominal SAT in humans has the highest content of preadipocytes compared to the VAT. More importantly, adipocyte stem and progenitor cells’ in vitro counterparts, here referred to as the adipose-derived stromal/stem cells (ASC), are depot-specific, with an inverse relationship between the adipogenic and immunogenic status: ASC from VAT are more pro-inflammatory in terms of cytokine secretion, with lower adipogenic potential [18]. We have previously suggested that stem cells and progenitor cells can regulate WAT expansion and the chronic inflammatory scenario of obesity in a WAT depot-dependent manner [4]. Indeed, proliferation of adipose precursors induced by a high-fat diet is regulated in a depot-dependent manner in mice [21,22,23]. Moreover, a subpopulation of murine adipose perivascular progenitor cells (platelet-derived growth factor receptor-beta positive, PDGFRβ^+^), termed “fibro-inflammatory progenitors” (FIPs; LY6C^+^PDGFRβ^+^ cells), regulates visceral WAT macrophage accumulation in mice fed a high-fat diet in a Toll-like receptor 4-dependent manner resulting in WAT dysfunction [24]. PDGFRβ+ perivascular cells are precursors of adipocytes in mice, both in inguinal and visceral WAT pads, with varying levels of the zinc finger protein 423 preadipocyte commitment factor (Zfp423) that distinguish subpopulations of adipogenic from inflammatory cells [25]. These authors also described that the frequency of adipogenic precursors and adipogenesis is depot-specific.

Recent efforts on single-cell level profiling of adipocyte progenitors and on clonal analysis of their in vitro counterparts (ASC) derived from different WAT depots of donors with a variety of metabolic phenotypes have clarified the understanding of ASC heterogeneity, their depot-dependent characteristics, and their contribution to the obesity scenario [20]. Vijay and coworkers [26] characterized different cell types that are WAT depot-specific or correlate with metabolic status (with or without type 2 diabetes) using single-cell RNA sequencing (scRNA-Seq) of WAT depots from obese donors. The authors identified progenitor cells with expression signatures that were dependent on their respective WAT depot. More importantly, a higher abundance of a subtype of preadipocytes was identified in individuals with hyperglycemia levels compared to those with normal glycemia. In addition, lymphatic-derived endothelial cells were more frequent in the VAT samples.

The intrinsic differences in ASC derived from VAT and SAT are also present in a non-obese state and are retained during obesity. The immunogenic factor bone marrow stromal cell antigen 2 (*BST2*) was identified as a marker of visceral ASC in a non-obese state in humans. However, the difference in *BST2* expression between subcutaneous and visceral ASC is more pronounced in obesity and with insulin resistance. In addition, in vitro-derived adipocytes from non-obese ASCs of VAT have lower gene expression of adipogenic markers and higher gene expression of immunogenic markers than those derived from SAT [27]. Raajendiran and collaborators [19] identified three adipocyte progenitor cell subtypes with distinct molecular patterns, but with similar adipogenic capacity. The characterization of the adipocyte progenitor cell subtypes was based on the expression of CD34 among CD31^−^CD45^−^CD29^+^ SVF cells. Interestingly, adipocytes derived from each progenitor subtype display distinct metabolic and endocrine phenotypes. Furthermore, adipocyte progenitors were more frequent in gluteo-femoral SAT than in abdominal SAT and VAT, and the frequency of adipocyte progenitor subtypes varies among donors with type 2 diabetes. ASCs showing depot-specific genetic [28], adipogenic, immunogenic, endocrine, and even extracellular vesicles [29] profiles are an interesting source of cells to decipher the cellular and molecular mechanisms that govern WAT physiology and dysfunction in a depot-specific manner.

The preferable expansion of gluteo-femoral SAT depots is typical of women and is associated with a lower risk of cardiometabolic dysfunction. On the other hand, VAT depot is expanded preferentially in men and is a predictor of cardiometabolic disease [30,31,32,33]. A recent study supported these clinical findings showing a depot- and sex-dependent adipose progenitor cell heterogeneity in mice [34]. We can speculate that ASCs derived from sex- and depot-targeted human tissue samples with their specific molecular signatures may be useful for evaluating and identifying the biological underpinnings of sex differences in WAT expansion in obesity and their relationships to metabolic health in men and women [35].

Adipocyte stem and progenitor cell heterogeneity and depot-dependent characteristics from adipose SVF have been widely explored at the single-cell level in murine-derived samples [36]. However, the extent to which the tissue architecture and composition of mouse adipose tissue resembles that of humans is still unknown and limits the translation of obesity-related findings from murine to human. Vijay and coworkers [26] have recently explored SVF heterogeneity in adipose samples derived from obese donors using single-cell transcriptomics. In addition, ASCs’ depot-dependent profile has been described for human WAT during the last decade [4,18,27]. Therefore, there is a need to specifically study human adipose tissue-related physiology and diseases in experimental systems engineered with human-derived cells with depot-specific profiles to mimic key morphofunctional properties.

### 2.2. Subcutaneous Adipose Tissue Layers as Adipose Tissue-Derived Stem/Stromal Cells Microenvironments

Human SAT is separated into two layers by a dense conjunctive tissue named fascia [37]. The superficial layer is located close to the dermis, having well-defined and compacted lobules, while the deep layer shows more loose and disorganized larger lobules [38]. The superficial layer of SAT supports properties already described for the entire tissue such as anti-inflammatory and regenerative, acting as a protective depot in metabolic syndromes [39,40]. This layer contains a higher number of small adipocytes, as well as larger lipid droplets compared with deep SAT, showing a high potential for adipogenesis [5,41,42,43,44,45] (Figure 1).

We recently analyzed superficial and deep layers in SAT non-obese samples. The SVF of the superficial layer showed the highest percentage of preadipocytes and a predominant presence of arterioles [5]. The adventitia layer of arterioles was previously described as a preadipocyte niche in SAT [46]. Furthermore, ASCs derived from the superficial layer have a significantly greater surface of lipidic droplets together with the highest number of unilocular cells and up-regulation of CEBPα and FABP4 genes [5], supporting a high adipogenic potential previously described for this layer.

On the other hand, the deep layer of SAT is correlated with high levels of inflammatory cytokines and adipokines, besides its disproportionate expansion observed in obese Caucasian males [47,48]. ASCs derived from the deep layer revealed the lowest levels of adipogenic and secretory capacities [5]. Monzon and collaborators [49] compared the lipolysis in adipocytes isolated from the deep and superficial layers and found an increase in lipolysis in the deep layer. 

### 2.3. The Stem/Progenitor Cells Derived from the Fascial System of Subcutaneous Adipose Tissue

SAT is inserted into the fascia to form a structural and functional continuity over the body [50]. Each fascia system at distinct tissues and organs operates independently, but at the same time is interdependent with the whole system [51].

Young rats have a primitive SAT, making them an ideal animal model for investigating adipose tissue origin. In this context, Su and collaborators [52] described the generation of adipocytes from the adventitia of blood vessels leading to the formation of primitive adipocytes lobules in the fascia. Zhang and collaborators [6,53] demonstrated that the superficial fascia of rats has a population of adipocyte progenitor cells capable of forming adipose tissue organoids with functional unilocular adipocyte-like cells.

In human SAT, the conjunctive extensions derived from the fascia are more prominent at the superficial layer, being named retinacula cutis [54]. The retinacula cutis of the superficial layer showed double positive staining for CD34 and CD31, revealing the presence of endothelial progenitor cells. Interestingly, Pref-1 staining was found exclusively in retinacula cutis and in adventitia of blood vessels while it was absent in adipose tissue itself [5]. Furthermore, ASCs derived from retinacula cutis showed the highest secretion in vitro for vascular endothelial growth factor (VEGF) compared with ASCs from both superficial and deep layers [5]. Recently, Ziegler and collaborators [55] showed an angiogenic genetic profile in the human fascia matrix, supporting our results with ASCs derived from retinacula cutis.

Interestingly, retinacula cutis revealed a continuity with the adventitia of blood vessels, being the adventitia niche more frequent in the superficial layer of SAT [5]. A previous study by our research group found that SAT samples from ex-obese subjects had a higher number and size of blood vessels and revealed a preferential location of these blood vessels close to the dermis [56]. These results were later associated with an increase in preadipocytes in SVF of ex-obese SAT samples [57].

### 2.4. The Dermis Can Be Stratified According to Its Fibroblasts Subpopulations

The dermis is a connective tissue located between the epidermis and the hypodermis. The dermis is composed of two different layers, a papillary dermis just below the dermo–epidermal junction and deeper at the reticular dermis. Fibroblasts are the most abundant cells in the dermis; they secrete and remodel the extracellular matrix (ECM) which allows the dermis to be a support tissue for the skin. There is an ECM signature of the different fibroblasts subpopulation [58]. The papillary dermis presents an important cellular density with more proliferative fibroblasts than the reticular dermis [59,60]. ECM of the papillary dermis present collagen fibrils loosely organized, thin unstriated fibrillar material, and proteoglycan aggregates [61]. The papillary fibroblast regulates hair growth and plays an indispensable role in re-epithelialization during wound healing [62]. The papillary dermis role is to interact physically and chemically through growth factors with the epidermis. The reticular dermis is thicker and presents an ECM with aligned collagen fibrils and a dense network of elastin [61]. Its role is to confer tensile strength to the dermis. The reticular fibroblast initiates healing by matrix production [63].

In addition to their role in ECM regulation, fibroblasts also interact with other cell types such as ASCs, located in SAT just beneath the reticular dermis. Haydont and collaborators [64] recently described a high adipogenic potential for a subpopulation of fibroblasts located at the dermo–hypodermal junction of the skin. The fascia has extensions originating from the dermis passing through the superficial layer and becoming looser and scarcer until reaching the deep SAT [54]. If dermo–hypodermal fibroblasts dwell at these extensions, one hypothesis is that these cells can infiltrate the SAT, and due to their plasticity, these fibroblasts can assume different behaviors according to the niche.

If fibroblasts can interact with ASCs modulating their behavior, ASCs can also do the same with fibroblasts, as suggested by in vitro assays.

ASC’s conditioned culture medium increased proliferation of dermal fibroblasts [65,66]. This proliferative effect of ASC’s conditioned culture medium in fibroblasts can be decreased by TGF-beta1 [67]; however, it does not interfere with the secretion of ECM; more specifically, the secretion of type I collagen and fibronectin are increased [65,67]. Interestingly, ASC’s conditioned culture medium can decrease the secretion of metalloproteinase, which can explain, in part, the increase of ECM secretion by fibroblasts [68]. Auxenfans et al. [69] also demonstrated that a 3D-reconstructed skin model co-cultured with ASCs increased thickness of the dermis, specifically the papillary dermis. In direct juxtacrine co-culture and in indirect paracrine co-culture, ASCs improved collagen maturation and metalloproteinase secretion compared to monoculture [70].

The beneficial effect of ASCs in dermis is even observed in altered conditions, such as keloid scar, since TGF-beta1-induced myofibroblast differentiation and human dermal fibroblasts’ function were inhibited by ASC’s conditioned culture medium [71]. In addition, Borrelli et al. [72] identified a subpopulation of ASCs positive for CD74 with enhanced antifibrotic effects. Dermal fibroblasts incubated with ASC’s conditioned culture medium from CD74 positive cells produced less collagen under TGF-beta1 stimulus compared to those incubated with ASC’s conditioned culture medium from CD74 negative cells. ASCs positive for CD74 may attenuate production of pro-fibrotic ECM components by fibroblasts and could promote improvement of detrimental histologic and biomechanical changes to skin following skin radiation injury.

To conclude, the scientific literature supports the crosstalk between dermal fibroblasts and ASCs that impact skin quality. Soluble factors secreted by ASCs seem to regulate the skin microenvironment according to the needs. Surprisingly, a recent study showed that diabetic microenvironment-preconditioned ASCs effectively strengthen the capacity against inflammation and modulate the progress of long-term T2D complications [73]. In addition, patients receiving fat grafting in subcutaneous areas exposed to radiation injury show improved cosmetic and functional skin outcomes, such as skin softness and pliability increase, volume restoration, hair growth improvement in areas of alopecia, and pain decrease [74]. It is suggested that endogenous stem and progenitor cells that reside within the SVF of adipose tissue could drive regenerative mechanisms by which fat grafting can slow or reverse skin radiation-induced fibrosis [75]. Indeed, fat grafts enriched with human CD34+CD146+ adipose-derived stromal cells enhance fat graft retention and vascularization and promote recovery of soft tissue after radiotherapy in mice [76]. However, there is a lack of research studies focusing on the specific interaction of ASCs with fibroblasts’ subpopulations, and more importantly, to what extent the ontogeny of fibroblasts and ASCs is correlated, since dermal fibroblasts can infiltrate the SAT.

## 3. Adipose Tissue 3D Models

ASCs are capable of reproducing in vitro their corresponding adipose tissue origin. However, 2D culture has severe limitations. Long-term cell culture is not possible using monolayers, limiting cell differentiation and disease models. More importantly, the adipose tissue microenvironment is complex showing cross-talk among adipocytes, pre-adipocytes, ASCs, endothelial and immune cells. The first strategy for adipose tissue 3D models had a focus on soft tissue reconstruction in the plastic surgery field. The understanding of the adipose tissue as an endocrine organ and obesity as an inflammatory chronic disease boosted these 3D models. Currently, the main concern is to recapitulate physiological functions of adipose tissue and its alterations during obesity development with corresponding exogenous stimulus and cellular components.

### 3.1. Adipose Tissue Engineering

**Figure 2:**
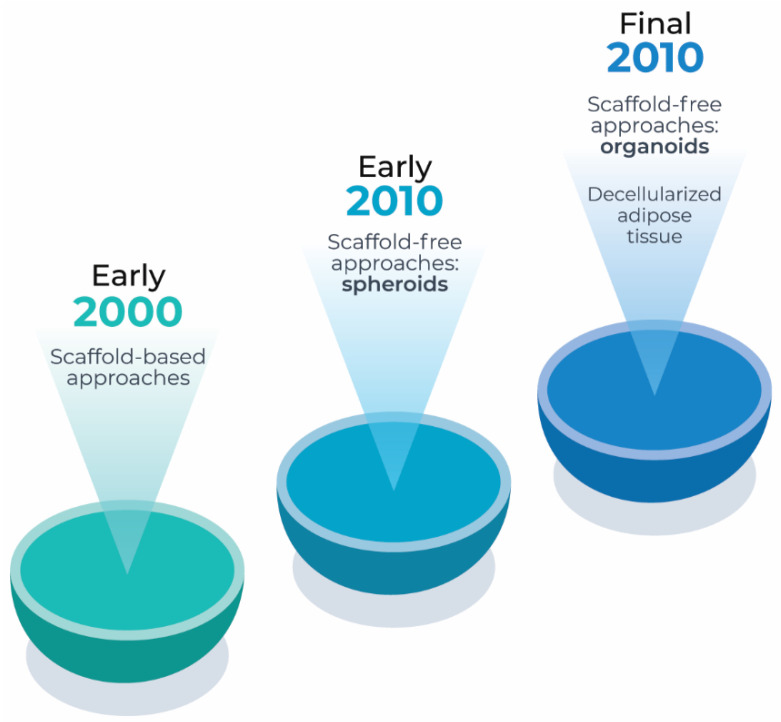
Timeline of adipose tissue 3D models.

In 1993, Langer and Vacanti [77] established the state of the art of tissue engineering based on the use of scaffolds to develop biologic substitutes for tissues and organs. The scaffold-based principle is to seed a cell suspension in a 3D polymeric scaffold to mimic the microenvironment of native tissues. The pioneers’ studies in adipose tissue engineering applied to soft tissue defects used similar strategies. In this sense, porous biodegradable polymer foams [78], hydrogels as injectable materials [79], and silk fibroin 3D scaffolds were explored [80]. Recent scaffold-based strategies are focused on the use of decellularized adipose tissue [81,82] (Figure 2). Decellularized adipose tissue can support human ASC viability [83,84,85,86], proliferation [83,84,85,87,88], and adipogenic differentiation [85,86,88,89,90]. In addition, it enables host cell infiltration and neovascularization after implantation [84,90,91].

Despite the success of the scaffold-based strategies for soft tissue reconstruction in animal models [81,92], this 3D culture approach does not favor cell–cell and cell–extracellular matrix interactions, cell differentiation, and organogenesis events [93], hampering their use as complex 3D disease models. Spheroids and organoids emerged as suitable 3D models for health and disease. In the absence of an adherent surface, stem and progenitor cells maximize their cell–cell adhesion which in turns guides extracellular matrix synthesis and cell differentiation, recreating complex 3D tissues and organs [94].

By definition, a spheroid is a spheroidal 3D structure constituted by mature, progenitor, or stem cells and extracellular matrix molecules. On the other hand, organoids must be formed from stem cells derived from healthy or pathological tissues, having at least one physiological function corresponding to the desired organ and not necessarily showing a spheroidal 3D structure (Table 1). However, most studies of adipogenic 3D models using spheroids still rely on the 3T3-L1 cell lineage (mouse preadipocytes) [95,96,97,98]. In fact, few articles explored the use of ASCs to form and maturate adipose tissue spheroids [99], and studies with organoid models are still recent [6,100].

### 3.2. Adipose Tissue In Vitro 3D Models for Obesity

Due to the chronic inflammatory scenario established during obesity, obese adipose tissue in vitro 3D models intend to mimic this inflammatory microenvironment using different approaches. Turner and collaborators [101] showed that under TNF-alpha stimulus, a proinflammatory molecule, 3T3-L1 spheroids increased their lipolysis function. Afterward, the same research group showed adipogenic maturation of human ASCs and 3T3-L1 spheroids under dietary fatty acids [102].

Fatty acids can also act as a proinflammatory stimulus. Recently, Pieters and collaborators (2022) treated human ASC embedded into hydrogel with fatty acids after adipogenic differentiation. The resulting adipocytes showed obese characteristics such as hypertrophy, increased lipolysis, and insulin resistance [103]. The phenomenon of lipolysis is commonly described in fat depots with more inflammatory properties, such as VAT [104].

Insulin resistance can also be mimicked in 3D adipose tissue models. ASCs were co-cultivated with HUVECs after adipocyte differentiation in a 3D silk scaffold. Only co-culture ASCs differentiated into adipocytes showed a decrease in triglycerides content which may suggest an increase in lipolysis [87]. This study revealed the importance of other cell components of adipose tissue microenvironment in the 3D models, since an isolated exogenous stimulus cannot replicate the complexity of cell–cell interaction.

Besides the relevance of endothelial cells, macrophage has a central role in the chronic inflammatory scenario established in adipose tissue in obesity [13,14]. A recent proteomic analysis revealed the up-regulation of proteins involved in carbohydrate metabolism and mitochondrial fatty acid beta oxidation pathway only in 3D co-culture of 3T3-L1 and macrophages [105]. Using a similar 3D co-culture model, Park and collaborators [106] showed a functional metabolic similarity to adipose tissue in diabetic mice. Similar results were found in a 3D co-culture model of 3T3-L1 and RAW264.7, a macrophage cell lineage, embedded in alginate beads compared with only co-culture cells showing insulin resistance markers [107]. Human ASCs or mouse 3T3-L1 and RAW264.7 were co-cultivated embedded in hydrogel as an anti-diabetic drug testing model. Human ASCs and mouse 3T3-L1 revealed different responses to the same group of drugs, highlighting the importance of interspecies variability [108].

In the chronic inflammation scenario established in an obese adipose tissue, the long-term presence of proinflammatory macrophages leads to an unsolved fibrosis microenvironment [109]. Rajangam and collaborators [110] established a 3D human ASCs spheroid model to mimic adipose tissue fibrosis. ASCs suspension was seeded in cell culture plates with immobilized FGF2 in well surfaces leading to an increase of TGF-beta1 secretion, a profibrotic molecule, at 5 days of spheroid culture. The authors discussed the use of this ASC spheroid model for testing antifibrotic therapies [110]; however, more importantly, this study opens up the possibility to increase the complexity of already published 3D models to mimic the chronic inflammation scenario of obesity.

### 3.3. Organoids as Recent Advances in Obese Models

Organoids have transformed the field of personalized medicine mainly due to their potential for predicting cellular and molecular events involved in the development of diseases [111,112].

**Figure 3:**
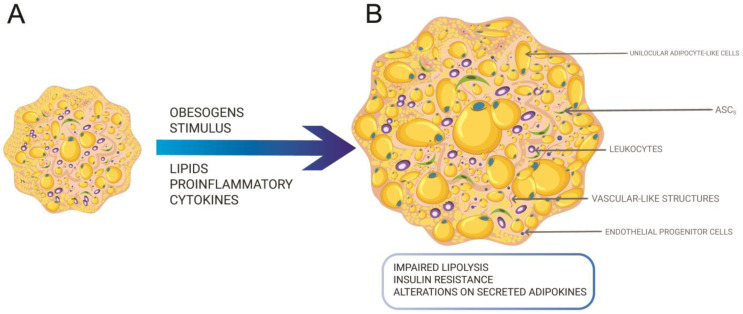
(**A**) SVF, a heterogeneous population of cells, can give rise to organoids when embedded into Matrigel or assembled with magnetic beads, showing endothelial progenitor and leukocytes cells besides ASC, preadipocytes, and adipocytes. (**B**) Adipose tissue organoids can give rise to an obese model under obesogens stimulus such as lipid and/or pro-inflammatory cytokines altering the functionality of organoids.

The SVF fraction comprises a heterogeneous cell population, showing the main cellular elements of SAT such as stem cells, endothelial and immune cells. In adipose tissue engineering, the organoid concept emerged with the use of SVF to form 3D constructs to recapitulate adipose tissue morphology and physiology, with a particular attention to vascular structures and adipocyte-like cells showing unilocular lipid vacuoles [100,113] (Figure 3A). In this sense, the pioneers’ study relied on magnetic levitation to assemble individualized cells into organoids. Daquinag and collaborators [113] explored mouse SVF capable of forming organoids showing vascular-like structures and a complex microenvironment composed of ASCs, endothelial cells, and leukocytes. In a subsequent study, spheroids were formed from human SVF and then transferred to Matrigel droplets maintained under adipogenic cocktail inductors [100].

In both studies, an external support (magnetic beads and Matrigel) was needed to maintain a 3D structure derived from SVF, distinct from ASC spheroids that secrete extracellular matrix components capable of sustaining their spheroidal morphology. Furthermore, these studies only showed functional improvements related to adipogenesis and angiogenesis [100,113]. Daquinag and collaborators [113] showed the presence of leukocyte cells; however, the presence of macrophages was not investigated. In a recent study, Ioannidou and collaborators [114] cultivated human SVF embedded in hydrogel to form adipose tissue organoids. The authors succeeded in mimicking in vitro weight gain by adding a natural mix of lipids to the cell culture medium. This addition led to altered adipocyte function attested by impaired lipolysis, insulin resistance, and an altered profile of synthetized adipokines (Figure 3B).

Interestingly, rat adipocyte progenitors were capable of migrating out of superficial fascia and generating adipose tissue organoids embedded in 3D hydrogel. These adipose tissue organoids derived from superficial fascia showed the majority of unilocular cells together with adipocyte functions involved with triglycerides metabolism and adipokine secretion [6]. One important limitation of the study of Zhang and collaborators [6] is the absence of endothelial cells due to the intrinsic nature of adipocyte progenitor cells. Furthermore, like the SVF organoids, the presence of a hydrogel was also required. Human adipose tissue organoids can also be obtained from ASC spheroids. To achieve this, the presence of stem cells and at least one physiological function must be attested [115,116]. In addition to the presence of unilocular cells, adiponectin and upregulation of key adipogenesis genes, clonogenic assays revealed the maintenance of ASCs inside spheroids [117].

Other studies using human ASC or SVF cells combined with human-derived hydrogels showed potential as a 3D in vitro model of adipose tissue [88,118]. Decellularized human adipose tissue-derived hydrogel supports the attachment, proliferation, and adipogenic differentiation of human ASC [88]. In addition, ASC remodeled the microstructure of the hydrogel, probably as a result of ASC attachment, migration, ECM production, and/or proteolytic activity—with MMP-2 (matrix metalloproteinase-2) as a potential regulator [88]. Bender and colleagues [118] validated an in vitro fat construct that combined cryopreserved human adipose tissue-derived SVF and a human blood-derived hydrogel (ObaGelTM). SVF cells in this culture system self-assemble into spheroids within one week of culture. Vascular-like structures are formed within these 3D constructs. Moreover, the hydrogel supports higher adipogenic differentiation of human SVF cells than bidimensional cultures, as well as higher glucose uptake, leptin secretion, and lipolytic activity [118]. These studies pave the way to biomanufacture a 3D construct to better recapitulate the biology of adipose tissue in an obesity context by combining decellularized adipose tissue and adipose SVF cells sourced from multiple adipose depots and derived from individuals with different body mass index and obesity degrees. Indeed, a recent study compared the physicochemical characteristics of decellularized adipose tissue hydrogel derived from lean and obese donors. Although hydrogels derived from both groups support ASC viability, proliferation, and differentiation, they present differences in their physical microstructure and proteomic profile. Importantly, decellularized adipose tissue hydrogels derived from obese donors retain an inflammatory microenvironment that is inherent to obesity [86].

To conclude, human ASC organoids compared with SVF organoids show some advantages including, but not restricted to: (1) ease of manipulation mainly due to the absence of hydrogel; (2) possibility of cell expansion and creating biobanks as a previous step to the organoids. However, one important disadvantage relies on the absence of endothelial cells. In this sense, alternative induction protocols must be tested. More importantly, mainly due to representing epigenetic alterations accumulated at stem and progenitor cells during obesity development, it would be interesting to obtain ASC organoids derived from obese donors. Obese organoids hold the potential of recapitulating the main cellular and molecular alterations which occurred during obesity development.

## 4. Perspective

Adipose tissue obese organoids will represent a powerful tool to modeling obesity besides testing potential drugs. However, besides their potential to revolutionize anti-obese drug discovery, organoids face challenges in their limited scale of production, absence of automation, costs, and reproducibility (Garreta et al., 2021) [119]. In addition, organoids are usually cultivated in a static environment, limiting their maturation capacity. Microphysiological systems based on microfluidics technology are known as organ-on-a-chip and can emulate several physiological parameters of tissues and organs. The convergence of organoid knowledge with advanced microphysiological systems [116] provides a more realistic tissue and organ microenvironment due to automation and reproducibility of an engineered system (Homan et al., 2019; Paek et al., 2019; Wang et al., 2020) [120,121,122]. Three-dimensional adipose tissue models started to be explored in microphysiological systems based on microfluid (McCarthy et al., 2020; Pope et al., 2020; Rogal et al., 2022; Compera et al., 2022) [123,124,125,126], and their integration with liver and pancreas will enable researchers to study obesity comorbidities such as nonalcoholic fatty liver disease and diabetes.

## 5. Conclusions

The diversity of WAT is reflected in its population of ASCs according to the adipose tissue physiology of each depot. Distinct layers of SAT reveal distinct ASC subpopulations and a possible integration with different subpopulations of fibroblasts located at the dermis, contributing together to skin and adipose tissue physiology. Recent advances in adipose tissue organoids opened an avenue of possibilities to recreate the main cellular and molecular events of obesity leading to a deep understanding of this inflammatory disease besides contributing to drug discovery. Obese organoids models will be more reliable when ASCs or SVF derived from obese donors are used, adding a personalized medicine context. Furthermore, 3D organ-on-a-chip will add reproducibility to these adipose tissue models contributing to their translation to the pharmaceutical industry.

## Figures and Tables

**Table 1:** Main aspects of spheroids and organoids.

	Spheroids	Organoids
Type of cells	Mature, progenitor or stem cells	Stem and progenitor cells
Self-assembly	Yes	In epithelial tissues is guided by the presence of a hydrogel
Morphology	Spheroidal	Diverse
Extracellular matrix	Strictly synthesized by cells	May contain exogenous components
3D architecture	Non-mimetic	Mimetic
Physiological function	Not necessarily	Yes
Morphogenesis	Only present when spheroid is derived from progenitor or stem cells	Present

## Data Availability

Data sharing not applicable.
